# A Soft Skin Adhesive (SSA) Patch for Extended Release of Pirfenidone in Burn Wounds

**DOI:** 10.3390/pharmaceutics15071842

**Published:** 2023-06-28

**Authors:** Eugene P. Chung, Jesse Q. Nguyen, Tobias Tellkamp-Schehr, Katja Goebel, Anita Ollek, Cliff Krein, Adrienne R. Wells, Eliza A. Sebastian, Anja Goebel, Svenja Niese, Kai P. Leung

**Affiliations:** 1 Combat Wound Care Group, US Army Institute of Surgical Research, JBSA, Fort Sam Houston, TX 78234, USA; 2 Department of Bioengineering, Rice University, Houston, TX 77005, USA; 3 Labtec GmbH, Raiffeisenstrasse 4, 40764 Langenfeld, Germanya.ollek@adhexpharma.com (A.O.); c.krein@adhexpharma.com (C.K.); s.niese@adhexpharma.com (S.N.); 4 MicRoN Core, Harvard Medical School, Boston, MA 02215, USA

**Keywords:** soft-skin adhesive, SSA, drug-in-matrix, patch, pirfenidone, burns, hypertrophic scars

## Abstract

As much as half or more of deep partial-thickness burn wounds develop hypertrophic scarring and contracture. Once formed, treatments are only minimally effective. Pirfenidone (Pf), indicated for treatment of idiopathic pulmonary fibrosis, is an anti-inflammatory and anti-fibrotic small molecule that potentially can be repurposed as a preventative against scarring in burn wounds. We present a drug-in-matrix patch with a soft skin adhesive (SSA) wound-contacting layer for multi-day drug delivery of Pf into burn wounds at the point of injury. Our patch construction consists of an SSA adhesive layer (Liveo™ MG7-9850, Dupont, Wilmington, DE, USA) for wound fixation, an acrylic co-polymer drug matrix (DURO-TAK 87-2852, Henkel, Düsseldorf, Germany) as the drug (Pf) reservoir, and an outermost protective polyurethane backing. By employing a drug-in-matrix patch design, Pf can be loaded as high as 2 mg/cm^2^. Compared to the acrylic co-polymer adhesive patch preparations and commercial films, adding an SSA layer markedly reduces skin stripping observed under scanning electron microscopy (SEM). Moreover, the addition of varying SSA thicknesses did not interfere with the in vitro release kinetics or drug permeation in ex vivo porcine skin. The Pf patch can be easily applied onto and removed from deep partial-thickness burn wounds on Duroc pigs. Continuous multi-day dosing of Pf by the patches (>200 μg/cm^2^/day) reduced proinflammatory biomarkers in porcine burn wounds. Pf patches produced by the manual laboratory-scale process showed excellent stability, maintaining intact physical patch properties and in vitro biological activity for up to one year under long-term (25 °C at 60% RH) and 6 months under accelerated (40 °C at 75% RH) test conditions. To manufacture our wound safe-and-extended-release patch, we present scale-up processes using a machine-driven automated roll-to-roll pilot scale coater.

## 1. Introduction

Pirfenidone (Pf) is an FDA-approved small molecule drug indicated for idiopathic pulmonary fibrosis with ongoing efforts to repurpose the drug for other fibrotic diseases [1]. Hypertrophic scars (abnormal dermal fibrosis) occur in 80% of the military personnel following burn injury [2]. Hypertrophic scars are raised, red, hard, itchy, and may cause abnormal sensations. Such pathological scarring can lead to severe functional impairment, psychological morbidity, and costly long-term healthcare [3]. Our groups and others have investigated the anti-inflammatory and anti-fibrotic properties of Pf when applied topically for preventing and mitigating burn wound-associated hypertrophic scars [4,5,6,7,8]. Hypertrophic scarring results from tissue damage and prolonged inflammatory and excessive fibrotic response, producing elevated levels of α-smooth muscle actin (α-SMA) and the excessive deposition of collagen during the repair process. When Pf in an ointment formulation was topically applied to mouse burn wounds, we observed a dose-dependent reduction of pro-inflammatory cytokines and α-SMA expression [9]. We also demonstrated that Pf inhibits human dermal fibroblast trans-differentiation to myofibroblasts, weakens the contractile machinery of activated dermal myofibroblasts, and decreases collagen deposition and fibrosis-related gene expression in vitro [10,11]. Additionally, we found that in vitro Pf treatment of lipopolysaccharide (LPS)-activated human neutrophils lessens chemotaxis, decreases production of pro-inflammatory reactive oxygen species and cytokines (TNF-α, IL-1β, and IL-6), and reduces neutrophil degranulation [12]. Moreover, Pf is reported to be well-tolerated by epithelial cells and mesenchymal stem cells at therapeutic doses [13].

Topical Pf exists commercially in Mexico at 8 wt% concentration in a pyrrolidone and carbomer-based gel under the brand KitosCell^®^. Clinical trials of KitosCell^®^ have reported significant reduction of scar formation from diabetic foot ulcers and pediatric burns, and it has additionally been marketed for acne treatment. However, KitosCell^®^ notably requires application three times daily which can be a barrier to treatment compliance [4,7,8,14]. Current reported drug formulations for Pf are primarily semi-solids including our previously reported ointment and poloxamer-based nanoemulsion gel [9,15,16]. These are spreadable, require frequent re-applications, and may be paired with a dressing that both prevents loss of vehicle to secondary contact and protects the underlying wound bed [17]. Dermal drug delivery has continued to rely predominantly on semi-solid formulations as next generation topical vehicles continue to be developed [18].

Adhesive patches applied to the skin are effective vehicles for multi-day drug dosing and have been used successfully for transdermal drug delivery, as well as for targeting underlying muscle [19]. However, the use of drug-loaded patches specifically for dermal delivery has been limited. One of the major barriers for application of adhesive systems directly over compromised skin is wound compatibility. Improper selection and usage of medical adhesives is a leading cause of skin injury in clinical settings, especially among elderly populations with fragile and thinner skin and those with skin conditions and sensitivities [20]. Two-part platinum catalyzed soft skin adhesives (SSA) have been adopted in wound-care dressing market as a wound safe adhesive material. This technology has been popularized by brands such as Safetac^®^ by Molnlycke due to their good adhesion but gentle release force that results in less skin stripping compared to other types of adhesive materials like acrylics [21,22,23].

Here, we present both the laboratory-scale development, manual fabrication, and the machine-driven automated pilot-scale production of an SSA patch with extended Pf release into burn wounds. The patch was constructed with a drug-in-matrix design which included a silicone SSA skin-contacting layer for wound fixation, an acrylic drug matrix as the drug (Pf) reservoir, and a flexible outermost protective polyurethane backing. Potential drug matrix materials were initially screened for high Pf loading capacity without drug crystallization. The effect of individual layer dimensions (thickness) on drug release was investigated in vitro, and permeation of Pf was assessed with intact burned porcine skin (ex vivo). We demonstrated that patch-formulated Pf maintained its biological activity in vitro with human dermal myofibroblasts and in vivo with a porcine model for deep partial-thickness burns. Lastly, we established the stability profile of laboratory-scale-produced patches and demonstrated excellent retention of drug content, biological activity, and other performance parameters. In summary, we showed a production-ready process to siliconize the popular monolithic patch design for effective drug delivery that can be used in transdermal treatments and applied onto compromised skin.

## 2. Materials and Methods

### 2.1. Animal Study Statement

Research was conducted in compliance with the Animal Welfare Act, the implementation of Animal Welfare regulations, and the principles of the Guide for the Care and Use of Laboratory Animals. The Institutional Animal Care and Use Committee approved all research conducted in this study. The facility where this research was conducted is fully accredited by the AAALAC International.

### 2.2. Animal Ethics Statement

The United States Army Institute of Surgical Research (USAISR) Institutional Animal Care and Use Committee (IACUC) approved all research conducted in this study (Animal Protocol A-17-018).

### 2.3. Pf Matrix Crystallization Screening

The maximum saturation solubility in various drug matrix materials was evaluated based on the microscopically observed crystallization of Pf over four weeks. Pf was added into either a silicone material (Liveo™ MG7-9850, Dupont, Wilmington, DE, USA) at 1 wt% or various solvent-based acrylate co-polymer materials (GELVA GMS 9073, DURO-TAK 87-2516, 87-2052, and 87-2852, Henkel, Düsseldorf, Germany) at 10–20 wt% Pf relative to the solids content of the adhesive materials. Prepared solutions were cast between 300–400 µm (thickness) and completely cured or dried, depending on the material. Crystallization for Pf was examined during production, after final preparation of the cast layer, or at 1, 2, 3, and 4 weeks post-production. This time frame was chosen to cover potential temperature deviations, for instance during shipment, as requested by the corresponding ICH guideline for stability testing [24]. During the 4-week post-production period, the samples were stressed and stored as follows: two days at 40 °C, two days at 4 °C, and three days at 25 °C in the style of a cyclic test design suggested by Carstensen and Rhodes [25] before being examined for crystallization once a week.

### 2.4. Laboratory Scale Patch Production: A Sequential Direct Coating Process

A polyurethane (PU) backing (PY-PT72AE, Lubrizol, Wickliffe, OH, USA) solution was prepared at 12.5 wt% in tetrahydrofuran (THF) for casting. Drug matrix casting solution was prepared by diluting the solids content of DURO TAK 87-2852 to 32 wt% using ethyl acetate. Pf was added at 15 wt% relative to the solids content of the DURO TAK 87-2852 solution and mixed with a SpeedMixer™ 150.1 FVZ dual asymmetric centrifuge (DAC, FlackTek Inc., Landrum, SC, USA). MG7-9850 was prepared by adding 1:1 ratio of MG7-9850 Part A and Part B and thoroughly mixing by DAC within 1 h of casting due to the pot-life after combining. All layers were sequentially cast over a paper carrier (RP-1K, Gardco, Columbia, MD, USA) described below and packaged with a siliconized release liner (Scotch-Pak 9709, 3M, St. Paul, MN, USA) over the SSA layer. All layer casting was performed with a Teflon-coated Digital Microm II casting knife (Gardco, Columbia, MD, USA) and an automatic drawdown film applicator (Byko-Drive XL, BYK, Wesel, Germany).

**Figure 1:**
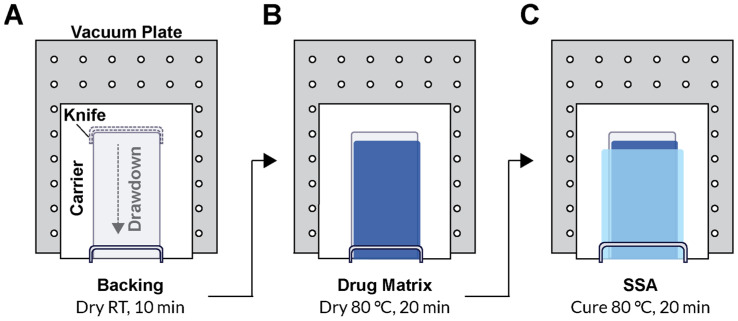
Sequential direct coating process schematic for laboratory scale production of SSA Pf patches. (**A**) Coating the PU backing and drying conditions. (**B**) Direct coating the drug matrix and drying conditions. (**C**) Direct coating the SSA with a wider caster and curing conditions. RT = room temperature.

To produce the patch, the paper carrier was first secured to the vacuum plate of an automatic film applicator. The casting knife was set manually to a gap width of 150 µm, and a 10 mm/s drawdown was performed on the PU backing solution. The solvent was evaporated for 20 min under continuous air flow. For the drug matrix, the casting knife gap width was typically set to 600 µm, although 400–1000 µm gap widths were also evaluated. The drug matrix casting solution was manually poured directly over the dried PU backing layer, and a 10 mm/s drawdown was performed. The drug matrix was dried at 80 °C for 1 h in a solvent-safe oven that produced a drug matrix significantly thinner than the original casting gap width. The backing and drug matrix on the paper carrier was removed from heat and allowed to cool to room temperature prior to addition of the SSA layer. The paper carrier was resecured to the vacuum plate of the automatic film applicator, and the mixed 2-part SSA was poured directly over the drug matrix layer above the PU backing layer. A wider casting knife than the one used for the previous two layers was used to draw down the final SSA layer. A 400 µm gap width over the previous layers was standard, but a range of 450–600 µm was also evaluated. The silicone layer was cured for 20 min at 80 °C. Lastly, a release liner was manually overlaid to the top of the SSA layer to produce the final assembly (Figure 1). Individual patches for dosing and samples for determining drug content were cut using a plot cutter (Maker 3, Cricut, South Jordan, UT, USA).

### 2.5. Visualization of Skin Stripping by Scanning Electron Microscopy (SEM)

Monolithic patches consisting of a backing and a DURO-TAK 87-2852 drug matrix and drug-in-matrix patches consisting of a backing, a DURO-TAK 87-2852 drug matrix, and a MG 7-9850 SSA skin contacting layer were prepared (refer to Section 2.4). Patches as well as commercial products, Tegaderm transparent film dressing (3M, St. Paul, MN, USA), and Mepitel One (Molnlycke Health Care AB, Gothenburg, Sweden) were applied on ex vivo full-thickness porcine skin. Samples were removed from the skin, and the adhesive sides were gold coated for 30 s using a sputter coater (DII-29010SCTR Smart Coater, JEOL, Tokyo, Japan) and imaged using a benchtop SEM (JCM-6000Plus, JEOL, Tokyo, Japan).

### 2.6. Pf Quantification

Pf was quantified by uHPLC (Ultra High-Performance Liquid Chromatography; Vanquish™ Flex Quarternary, Thermo Scientific, Karlsruhe, Germany) equipped with an INTERSIL ODS2 (150 Å, 5 μm, 4.6 mm × 250 mm) C18 column (USAISR research site method). Mobile phase consisted of 65:35 ratio at 0.02 M, pH 2.5, KH_2_PO_4_ in water:acetonitrile in isocratic mode with a flow rate of 1 mL/min and an injection volume of 20 µL. The temperature was maintained at 30 °C by air circulation in the column compartment, and Pf was detected at a wavelength of 310 nm with a retention time of approximately 9 min. The concentrations of unknowns were determined by comparing against a standard curve of Pf ranging from 3.9 to 500 µg/mL.

### 2.7. Patch Area Weight

Layer and total patch area weight was determined by preparing 0.5″ diameter samples across the dried/cured layer and massed on a microbalance. The area weight of the underlying layers from a given batch is measured and subtracted from the total layer assembly to determine the area weight of the drug matrix and SSA layers. Area weights of the patch are reported in mg/cm^2^.

### 2.8. Patch Drug Loading

Drug loading of Pf was determined by preparing 0.5″ diameter samples across the prepared patch. Pf was extracted into 10 mL of methanol in a glass vial for 48 h while rocking. Pf concentrations were quantified by uHPLC (refer to Section 2.6), and loading was reported in µg/cm^2^.

### 2.9. In Vitro Release Test

In vitro release tests (IVRT) were performed on a vertical diffusion cell (Vision^®^ Microette^™^, Hanson, Chatsworth, CA, USA) equipped with an automated sampling system (USAISR research site method). Diffusion cells had an effective diffusion area of 1.77 cm^2^ and a donor chamber height of 1 mm. Donor chamber was separated from receptor chamber with a regenerated cellulose membrane of MWCO 12–14 kD (Repligen, Waltham, MA, USA). Receptor chamber was 6.7 mL in volume and filled with 1× phosphate-buffered saline (PBS) at pH 7.4 and maintained at 32 °C using a recirculating water bath. In total, 500 μL samples were taken at 1, 2, 3, 4, 6, 8, 12, 18, and 24 h which involved a 1 mL flush followed by a sampling event. The Pf concentration in all samples was quantified by uHPLC (refer to Section 2.6).

### 2.10. In Vitro Permeation Test in Ex Vivo Porcine Skin

Duroc skin was harvested with a 750 µm collection thickness using a dermatome device. Deep partial-thickness burns (100 °C for 13 s) were applied on freshly collected skin using a 1.5″ diameter burn device (described in Section 2.11). Both intact and burned skin were applied to a vertical diffusion cell (Vision^®^ Microette^™^) with automated sampling. The receptor chamber was filled with 6.7 mL of 1× PBS with 0.05% gentamycin to prevent microbial growth and set to 32 °C. Pf patches were applied over the skin, and the receptor was sampled at 0, 1, 4 6, 12, 18, 24, 36, and 48 h. Pf concentration in all samples was quantified by uHPLC (refer to Section 2.6).

### 2.11. Porcine Model of Deep Partial-Thickness Burn Wounds

The method for establishing deep partial-thickness (DPT) burn wounds was adapted from the model described in Nguyen et al. [26]. A total of 24 3.0 cm-diameter deep partial-thickness (DPT) burn wounds that were position-matched were created on the mid region of the dorsum of 6 female red Duroc pigs, each weighing 45–65 kg. The total wound area including controls was less than 10% total body surface area (TBSA). The TBSA calculation was based on the Meeh’s formula (A = 10 × W2/3, A = area in cm^2^, 10 is a constant, and W = body weight in grams).

Prior to wounding, the pigs were anesthetized, and the placements of burn wounds and controls were traced with a skin marker. Burn wounds were created with a burn device designed by our group consisting of a 3.0 cm-diameter brass cylinder attached to a heat-insulated Teflon handle to allow safe handling by the operator. The burn device is electronically controlled by a J-type thermocouple embedded inside the brass cylinder. DPT burn wounds were created by applying the brass cylinder to the skin at 100 °C with a force of approximately 929.3 g for a total pressure of 131.36 g/cm^2^ as measured by an integrated force meter for 22 s. DPT burn depth was determined by assessing the degree of apoptosis as measured by terminal deoxynucleotidyl transferase dUTP nick-end labeling (TUNEL) stains of tissue cross-sections.

Pf loaded and vehicle patches were applied by first removing the clear release liner exposing the silicone face which was placed onto the wound. Once the patch was applied, the paper liner was removed, leaving behind the patch adhered to the wound. Pf- and vehicle-treated wounds were position-matched along the midline on the mid region of the pig’s dorsum to allow direct comparisons of treatment types. Tissues from wounds treated with Pf or vehicle patches were collected after the burn on post-operative (burn) day (POD) 7. From each pig, four 10 mm circular biopsies were collected from each wound (Pf patch or vehicle patch treated) from the epidermis to the entire dermis layer. Tissue samples isolated for total RNA sequencing were placed in cryotubes suspended in RNAlater (Thermo Scientific, Waltham, MA, USA) and stored at 4 °C. Following creation of burn wounds and excision of tissue on designated days, the wounds were dressed with Xeroform (Covidien, Mansfield, MA, USA), cotton gauze, Ioban (3M Health Care, St. Paul, MN, USA), and a fabric vest (DeRoyal, Powell, TN, USA).

#### 2.11.1. Residual Drug after In Vivo Application

For in vivo studies, patches were produced as described in Laboratory Scale Patch Production (refer to Section 2.4). Patches were applied during the 14 days post-burn for either 2, 3, or 7 days of continuous wear time. Used patches were recollected, and residual Pf was extracted into 40 mL of methanol for 48 h while rocking. Pf concentration was quantified by uHPLC (refer to Section 2.6) and compared to the known loading amount to determine the percent residual drug remaining after application.

#### 2.11.2. In Vivo Tissue mRNA Expression

Total RNA extraction was performed as described by Nguyen et al. [26]. Briefly, each biopsy sample was transferred to 5 mL of TRIzol (Life Technologies, Carlsbad, CA, USA) and homogenized using the T25 ULTRATURRAX at 20,000 rpm (IKA, Staufen, Germany). RNA was extracted with 1 mL chloroform. The RNA was purified using an RNEasy Mini-Kit (Qiagen, Germantown, MD, USA) with a QIAcube extraction robot (Qiagen, Germantown, MD, USA). Genomic DNA was removed by treatment with DNAse I (Qiagen, Germantown, MD, USA). The RNA was quantified by NanoDrop One (Thermo Scientific, Karlsruhe, Germany)

Quantitative RT-PCR was performed as described by Karna et al. [27]. Briefly, quantitative real-time PCR was performed using the SYBR green master mix (Bio-Rad, Hercules, CA, USA) with specified primers (Supplemental Table S1) and was analyzed using the StepOne System (Applied Biosystems, Waltham, MA, USA). Expression of mRNA in wound tissues was determined by 2^−ΔΔCt^ method and normalized by metal regulatory transcription factor 1 as the housekeeping gene. Assay was performed in technical triplicates.

### 2.12. Revised Laboratory Scale Patch Production: A Bilayer Laminate Process

In preparation for scale-up production, laboratory scale processes were revised from a sequential direct coating process to a bilayer laminate process. The drug matrix solutions were coated with a double screw coating knife PG-031-200 (Thierry, Stuttgart, Germany) and a target area weight of 12 mg/cm^2^ on the siliconized side of the process liner (PET 78GY6, Loparex, Forchheim, Germany) and dried at 30 °C for 10 min, followed by 60 °C for 30 min in a drying oven. The dried drug matrix was then laminated against a carrier-loaded PU backing (PU4142, Gerlinger, Netzschkau, Germany). After equilibration for at least 24 h, the process liner of the drug matrix was carefully removed at a 180° angle, and SSA was coated directly over the drug matrix with a double screw coating knife (PG-031-250) set at a target area weight of 10 mg/cm^2^ and cured at 100 °C for 10 min. The cured SSA was laminated with the release liner (LDPE 74000, Loparex, Forchheim, Germany).

### 2.13. In Vitro Permeation in Human Skin

The in vitro permeation study was executed according to the EMA-Guideline on quality of transdermal patches (Labtec research site method). Unidentified human full-thickness skin (living donations from patients undergoing plastic surgery) was fixed between the donor and acceptor compartments on a modified vertical static Franz cell (5 mL) set to 32 °C with a permeation area of 0.82 cm^2^. The receptor compartment was filled with 5 mL of phosphate buffer (pH 6.8) containing 0.1% *w*/*v* of NaN_3_ as the preservative. Patches were fixed by a stainless-steel grid for better adhesion during the experiment. An autosampler (Vision AutoPlus, Hanson, Chatsworth, CA, USA) was used for sampling collection and volume replacement of the acceptor medium in the permeation cell. After the runtime of 72 h of the permeation experiment, Pf was extracted from the patch after removing from the human skin to determine the extent of residual Pf present in the patch and the amount of Pf deposited in the skin and present in the receptor. The determination of the amount of Pf in the samples was done by HPLC with UV-detection using a Intersil ODS–2 C18 150 × 4.6 mm (150 Å, 5 µm) column. The mobile phase consisted of an isocratic mixture of 0.02 M KH_2_PO_4_ buffer/acetonitrile (68:32, *v*/*v*). The run time was 7 min, and the injection volume was 20 µL, with a column oven temperature of 30 °C, a flow rate of 1.0 mL/min, and a detection wavelength of 220 nm.

### 2.14. Stability Assessment

Samples of the drug containing patch and the vehicle prepared by the revised laboratory patch production (Section 2.12) were punched from the produced laminates and randomized depending on the analytical test at start (T0), after 1 month (T1), 3 months (T3), 6 months (T6), and 12 months (T12) storage at 25 °C at 60% RH and 1 month, 3 months, and 6 months storage at 40 °C at 75% RH.

#### 2.14.1. Peel Force

The determination of peel strength of Pf transdermal systems was performed by means of a tensile-compression testing machine (Zwicki 2.5 kN, ZwickRoell, Ulm, Germany) on polycarbonate plates (LexanTM, Tekra, New Berlin, WI, USA) using a 10 N load cell. Samples were cut to a size of 15 × 50 mm, the release liner was removed, and the sample was placed on the polycarbonate plate with the backing facing upwards. Adhesion was facilitated by a 2 kg rubberized steel roller. The plate was installed into the tensile-compression testing machine and the patch was removed in a 90° angle with a speed of 300 mm/min.

#### 2.14.2. Residual Solvents

The determination of residual solvents was performed at start (T0) of the stability study. Testing was carried out using a GC-FID (Agilent, Santa Clara, CA, USA) with a Multi-Purpose Sampler. A ZB 624 column (60 m, 3 µm, 0.53 mm inner diameter, Agilent) was used with hydrogen as mobile phase operating in constant flow mode at 4.5 mL/min. Samples were incubated at 150 °C for 60 min and injected at 230 °C. Then, 1000 µL was injected running a temperature program as follows: 2 min at 35 °C, increasing to 70 °C with 4 °C/min, subsequently increasing to 230 °C with 20 °C/min, and holding at 230 °C for 6.25 min. The whole cycle time of the method was 45 min.

#### 2.14.3. Microbiology Testing

The determination of sterility was performed at start (T0) and T6 of the stability study (under normal and accelerated conditions). Six Pf patches for each timepoint were assayed to determine their sterility. Trypticase Soy Broth (TSB) was used as the growth media. After removing the backing, each patch was placed in a 50 mL conical tube containing 10 mL sterile TSB. For sterility testing, three of the tubes that contained the patches received no challenge bacteria. As the media control, the other three tubes that contained the test patches received 10 µL (at 1 × 10^6^ colony-forming units per ml) of a challenge organism of *Staphylococcus aureus*, *Pseudomonas aeruginosa*, and *Candida albicans*, respectively. All six tubes were incubated aerobically at 35 °C in a shaker incubator for 7 days. Turbidity of the culture media was the endpoint for microbial growth.

#### 2.14.4. Revised Method for Drug Content and Degradation Products

The Pf content was determined via HPLC-DAD (Agilent/Thermo) using a Kromasil C18 250 × 4.6 mm (5 µm) column (Labtec research site method). The mobile phase A consisted of a mixture of 0.02 M KH_2_PO_4_ buffer/mobile phase B (95:5, *v*/*v*), and the mobile phase B consisted of acetonitrile/methanol (22:13, *v*/*v*). The percentage of mobile phase B was gradually increased to 30% over 10 min, held at 30% for 12 min, increased to 75% over a period of 5 min, and subsequently increased to 95% over 10 min. It was then decreased gradually to 0% for 3 min and held for another 5 min (total run time 45 min).

The injection volume was 15 µL, with a column oven temperature of 40 °C, a flow rate of 1.0 mL/min, and a detection wavelength of 220 nm.

#### 2.14.5. Dissolution/Drug Release

Dissolution tests were performed using a USP apparatus 6 (rotating cylinder, Hanson, Chatsworth, CA, USA) with NaH_2_PO_4_/phosphoric acid buffer at pH 4.5 as dissolution medium. The dissolution test was carried out at 32 °C in a 900 mL vessel at a rotation speed of 100 rpm. The samples were die-cut to 6 cm^2^ and adhered to the cylinder using double-sided adhesive tape. Samples of 5 mL were taken at 4 h, 18 h, and 48 h, and the medium was replaced. The samples were evaluated using a HPLC-DAD system (refer to Section 2.13) with a 50 µL injection volume.

#### 2.14.6. In Vitro Activity of Pf Patches in Human Dermal Myofibroblasts

The determination of the biological activity of Pf and vehicle patches was performed at start (T0), T6, and T12 of the stability study. To determine the biological activity of Pf patches, normal human dermal fibroblasts (NHDF) maintained in complete media (DMEM, 10% FBS, 1% penicillin-streptomycin) and cultured within 8 passages were used. Cell assays were performed as previously described [10]. For western blot assays, cells were seeded in complete media and grown for at least 24 h before serum starvation in serum-free media (SFM) with 1% penicillin-streptomycin. After 24 h in SFM, cells were treated with 10 ng/mL TGF-β1 with or without Pf (0.5 mg/mL). Fresh Pf solution dissolved in 1× PBS was compared with Pf released from patch preparations into PBS after extraction for 48 h at 37 °C. The concentration of fresh and released Pf was verified by uHPLC after solutions were filter sterilized (0.22 μm). TGF-β1-stimulated NHDF treated with fresh Pf or Pf extracted from Pf patches were directly lysed on the culture plate in Pierce RIPA buffer supplemented with Pierce EDTA-free protease inhibitors mini tablets. Plates were then scraped to collect whole cell lysates before samples were clarified by centrifugation at 14,000 RPM for 15 min at 4 °C. Protein concentration was quantified using the Pierce BCA assay. Immune detection of proteins was performed using the Simple Wes capillary-based Western blot technology as described previously (31) (Protein Simple, San Jose, CA, USA). Briefly, samples were adjusted to equivalent protein concentration using provided sample buffer; four-part protein sample was mixed with one part provided 5× master mix (Protein Simple) containing fluorescent molecular weight markers and DTT (40 mM). Samples were heated at 95 °C for 5 min prior to loading into supplied microplate. Primary antibodies for α-SMA (1:20) and GAPDH (1:500) were diluted in provided antibody diluent and added to the microplate along with provided HRP-conjugated secondary antibody, chemiluminescent substrate, and wash buffer. The automated capillary system, Simple Wes, separated the proteins by electrophoresis, immobilized the proteins, and then performed immunoblotting in the same capillary. Protein quantification was performed using the Compass software for Simple Western (v3.0.9; Protein Simple).

### 2.15. Roll to Roll Pilot-Scale Patch Production

For pilot-scale production, patches were manufactured on a semicontinuous roll-to-roll coater (Smartcoater SC-18, Coatema, Dormagen, Germany) equipped with a 400 µm slot-die in a two-step process. The drug matrix casting solution was prepared as in the laboratory scale. It was then cast on a process liner (PET 78GY6, Loparex, Forchheim, Germany), dried in stages in the different dryer zones at temperatures between 30 °C and 120 °C, and laminated against a commercial PU backing (PU4142, Gerlinger, Netzschkau, Germany) supported with a biaxially oriented polypropylene (BOPP) carrier. This monolayer was then wound up to generate the first patch roll stock. In the second coating step, the process liner was delaminated in a 180° angle to expose the drug matrix for coating of the SSA layer. Parts A and B of the SSA were fed through a static mixer and coated on the drug matrix monolayer, followed by a rapid curing of the SSA in a temperature gradient with a maximum of 110 °C. The resulting bilayer was then laminated against a release liner (LDPE 74000, Loparex, Forchheim, Germany) and wound up as the final patch roll stock. Before further processing, the BOPP carrier was carefully removed.

### 2.16. Statistical Analysis

For in vitro release tests, a two-way ANOVA (time and matrix thickness as factors) was conducted with a Bonferroni post-test compared to the thinnest formulation (* *p* < 0.05, ** *p* < 0.01). For in vivo mRNA expression levels, statistical significance was determined by paired *t*-test assuming Gaussian distribution with a two-tailed test of significance (* *p* < 0.03). For in vitro biological activity of patch-extracted Pf and vehicle controls, statistical significance was determined by one-way ANOVA with a Bonferroni post-test (* *p* < 0.05).

## 3. Results

### 3.1. Pf Matrix Crystallization Screening

Drug matrix materials were screened by evaluating the saturation solubility of Pf in commercially available adhesives (Table 1). DURO-TAK 87-2852 is a pressure sensitive adhesive (PSA) composed of an acrylate copolymer that demonstrated the highest solubility for Pf based on microscopic observation of crystallization. When loaded at 15 wt% relative to the solids content of the adhesive, Pf did not crystalize in a prepared drug matrix layer that was stressed under changing temperature conditions through the four weeks of observation. Furthermore, increasing the loading to 16.5 wt% or higher resulted in crystallization within one week of storage or less. Other PSAs demonstrated a range of saturation solubilities, from insoluble during casting solution preparation (DURO-TAK 87-2516) and up to 12.5 wt% loading (GELVA GMS 9073) of Pf with no crystallization observed within the prepared matrix at four weeks of storage. MG 7-9850 soft skin adhesive (SSA) was not a suitable drug matrix since Pf is practically insoluble (<1 wt%) during the preparation and mixing of the two-part solution.

### 3.2. Patch Construction and Pf Release

Pf was further formulated in a drug-in-matrix patch design comprised of three layers. In the down-selected DURO-TAK 87-2852 drug matrix, Pf was loaded at 15 wt% relative to the adhesive solids content. This was supported by a flexible PU backing, and MG 7-9850 SSA was added as a skin contacting adhesive layer. We evaluated how to effectively add MG 7-9850 onto the classic monolithic patch design and noted that laminating separately prepared SSA and drug matrix layers did not result in effective bonding. After applying and removing patches that were assembled through lamination, the SSA layer delaminated and was left on the test substrate (data not shown). Switching to a direct-coating process that involved casting MG 7-9850 directly over the drug matrix followed by high temperature curing resolved patch failure from delamination. As a final laboratory scale process (ISR site), patches were constructed by sequential direct coating over a process liner, initially casting the PU backing, followed by the drug matrix, and lastly the SSA layer (refer to Section 2.4). (Figure 1A–C).

**Figure 2:**
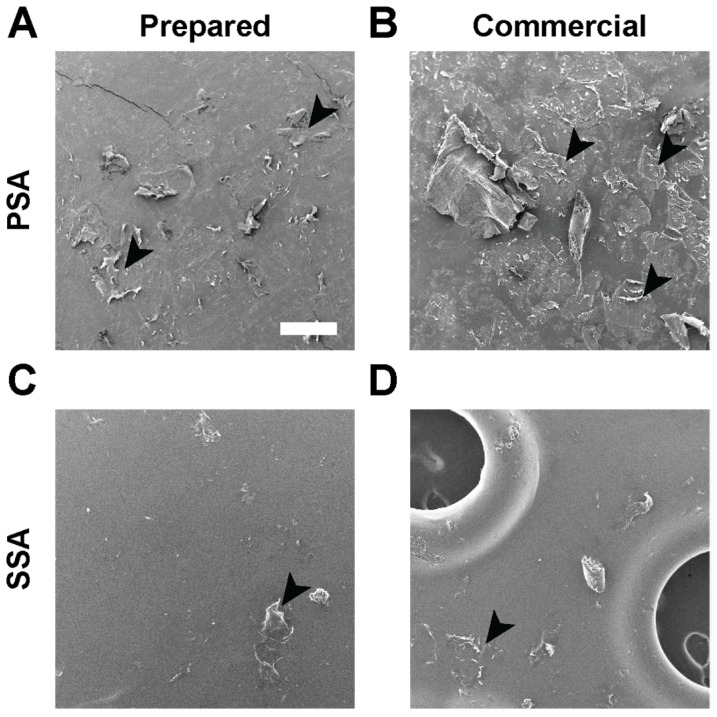
SEM skin stripping visualization on PSAs and SSAs surfaces. (**A**) DURO-TAK 87-2852 PSA skin contacting layer; (**B**) Tegaderm commercial transparent film dressing; (**C**) MG 7-9850 SSA skin contacting layer; and (**D**) Mepitel One commercial wound dressing. Arrows indicate stripped epithelial cells on the adhesive surface. Scale bar = 500 μm.

When omitting the MG 7-9850 layer and using DURO-TAK 87-2852 directly as the skin contacting adhesive, SEM visualization demonstrates higher skin stripping (Figure 2). This difference in adhesive performance was consistent when evaluating commercially available products that use a PSA (Tegaderm, 3M) or SSA (Mepitel One, Molonyke) skin contacting layer.

**Figure 3:**
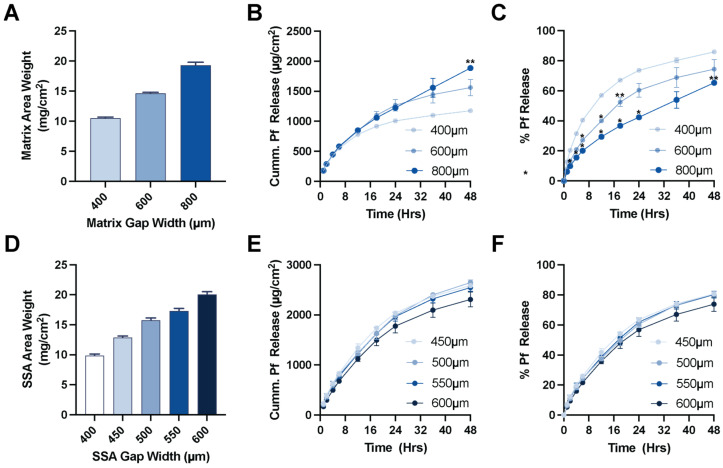
Effect of patch layer thickness of Pf release. (**A**) Drug matrix coating gap width and effective area weight (mean ± SD, *n* = 9). (**B**) Drug matrix coating gap width and cumulative Pf release profile (mean ± SD, *n* = 2, ** < 0.01 compared to the 400 μm matrix). (**C**) Drug matrix coating gap width and % release of Pf (mean ± SD, *n* = 2, * < 0.05, ** < 0.01 compared to the 400 μm matrix). (**D**) SSA coating gap width and effective area weight coated over a constant drug matrix (mean ± SD, *n* = 3). (**E**) SSA coating gap width and cumulative Pf release profile (mean ± SD, *n* = 2). (**F**) SSA coating gap width and % release of Pf (mean ± SD, *n* = 2).

The effect of drug matrix thicknesses on the in vitro release of Pf was evaluated by testing a range of coating gap widths. For a DURO-TAK 87-2852 matrix loaded with 15 wt% Pf, increasing the coating gap width from 400 μm to 800 μm proportionately increased the area weight of the dried drug matrix from 10.5 ± 0.2 mg/cm^2^ to 19.3 ± 0.5 mg/cm^2^ (Figure 3A). Cumulative release of Pf up to 12 h was comparable between the different drug matrix thicknesses (Figure 3B). Moreover, there was an inverse relationship for % Pf released and drug matrix thickness (Figure 3C). However, given the increasing Pf content in thicker drug matrix preparations, these demonstrated longer release profiles. The 400 μm preparation had a cumulative Pf release of 780.3 ± 11.0 µg/cm^2^ after 12 h accounting for 57% release of loaded drug (Figure 3D). While the 800 μm preparation had comparable 12 h cumulative Pf release of 850 ± 1.1 µg/cm^2^, this only accounted for 29.4 ± 0.0% release, where it continued to demonstrate near linear release kinetics out to 48 h. A two-way ANOVA with Bonferroni post-test comparisons against the thinnest 400 µm drug matrix show significantly slower percent release of Pirfenidone over time with thicker drug matrixes.

We further evaluated how increasing the casting thickness of MG 7-9850 over a constant drug matrix (23.1 ± 0.5 mg/cm^2^) affected the patch performance. Coating directly over the underlying backing and drug matrix using a wider caster and increasing gap widths from 400 to 600 μm gap width proportionately increased the effective area of SSA from 9.9 ± 0.3 mg/cm^2^ to 20.1 ± 0.4 mg/cm^2^ (Figure 3D). Increasing SSA layer thicknesses exhibited minor effects on the cumulative release kinetics from the patch (Figure 3E,F). However, SSA layer thickness was critical for the adhesive performance. SSA materials require a minimum thickness to achieve targeted adhesive qualities where SSA layers prepared with an effective area weight of 10 mg/cm^2^ or less resulted in delamination of the silicone when constructed through sequential direct coating (Supplement Figure S1).

### 3.3. Pf Permeation in Duroc Skin

**Figure 4:**
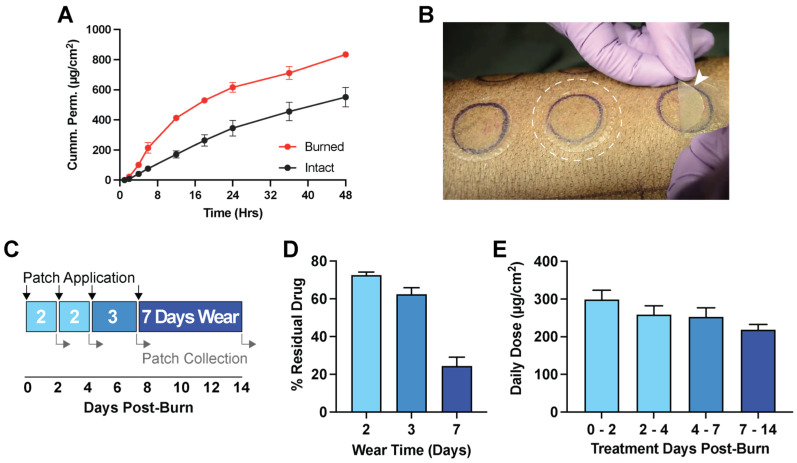

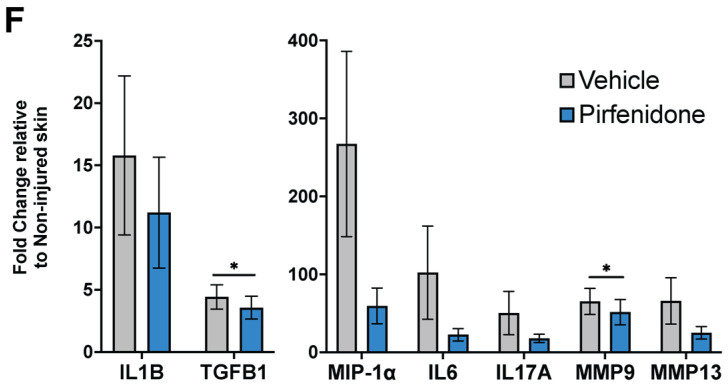
Pf permeation in burned Duroc skin. (**A**) IVPT of Pf through intact and burned ex vivo Duroc skin (mean ± SD, *n* = 3). (**B**) Image of patches applied on DPT burns in vivo. Arrow shows patch during application and dashed circle highlights an applied patch. (**C**) Timeline for Pf patch application and collection from DPT burns in vivo. (**D**) % residual drug remaining in collected patches after 2, 3, and 7 days of wear time (mean ± SD, *n* = 23–32). (**E**) Daily average of Pf permeation between POD 0–14. (**F**) mRNA expression of inflammatory markers in wounds biopsied on POD 7 (mean ± SEM, *n* = 4, * *p* < 0.03).

A 2 mg/cm^2^ loaded Pf patch was produced for further evaluation of drug permeation in Duroc skin. Using skin harvested from Durocs, we observed that Pf can readily permeate through ex vivo intact full-thickness skin (Figure 4A). After 24 h, a cumulative permeation of 345.6 ± 89.6 μg/cm^2^ was measured in the receptor, and after 48 h, 551.2 ± 112.5 μg/cm^2^. However, if the barrier function and integrity of ex vivo skins were compromised by emulating a deep-partial thickness burn, significant increases in cumulative permeation were observed. In ex vivo burned skin, Pf had a cumulative permeation of 616.5 ± 57.3 μg/cm^2^ after 24 h and 834.7 ± 30.1 μg/cm^2^ after 48 h.

We further evaluated the permeation of Pf patches applied to DPT burns in vivo (Figure 4B), determined by the residual drug present in recovered patches after 2, 3, or 7 days of wear time (Figure 4C). The patches produced in this study had an average drug loading of 2.05 mg/cm^2^ with less than a 5% relative standard deviation of discs sampled across the larger area of each produced sheet (Supplemental Figure S2). The total patch area weight was also consistent across the multiple batches produced, with an average area weight of 29.5 mg/cm^2^ with a relative standard deviation of <5% across the batches produced. We observed a time-dependent depletion of drug from our patches. For 2, 3, and 7 days of wear time, we measured in the collected patch 72.5 ± 13.4%, 62.4 ± 19.6%, and 24.5 ± 22.4% residual drug remaining, respectively (Figure 4D). These values were extrapolated to determine the effective average daily Pf dose deposited into burn wounds across the wear time for POD 0–2 (298.5 ± 139.2 μg/cm^2^), POD 2–4 (258.6 ± 132.5 μg/cm^2^), POD 4–7 (253.0 ± 134.4 μg/cm^2^), and POD 7–14 (219.0 ± 64.8 μg/cm^2^) (Figure 4E).

The anti-inflammatory activity of Pf was assessed by mRNA expression of pro-inflammatory markers in treated (Pf or vehicle patch-treated) DPT wounds biopsied at POD 7 post-burn. We observed a significant lower expression of MMP-9 and TGF-β1 in Pf-treated relative to vehicle-treated wounds (Figure 4F). When wounds were treated with a patch vehicle, burn wounds had a 65.2- and 4.4-fold increase in MMP-9 and TGF-β1 expression relative to non-injured skin, respectively. In contrast, 51.6- and 3.6-fold increases in MMP-9 and TGF- β1 expression were observed in wounds treated with Pf loaded patch (Figure 4F). The differences were statistically significant (*p* < 0.03; Figure 4F). Although not statistically significant, we also observed similar trends showing lower CCL3, IL-1β, IL-6, IL-17A, and MMP13 expression in DPT wounds treated with Pf compared to vehicle controls.

### 3.4. Pf Permeation in Human Skin

Next, we evaluated the permeation of the Pf patch using the ex vivo human skin. These Pf patches were prepared by the revised laboratory scale patch manufacturing process. Instead of manually casting the PU backing layer, these Pf patches were fabricated using a premade PU backing that is commercially available as a roll stock (Section 2.12). Additionally, SSA layers of different thicknesses were prepared in these Pf patches. The thickness of the premade backing did not cause significant changes in cumulative permeation of Pf (Supplemental Figure S3A). Changing the thickness of the SSA layer also did not have major effect on permeation (Supplemental Figure S3B).

**Figure 5:**
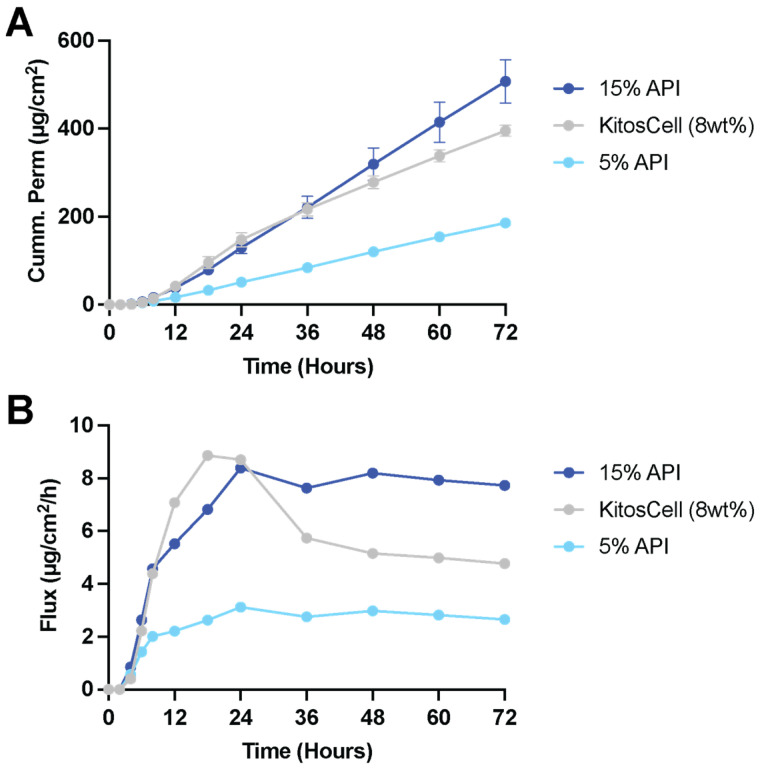
IVPT assessment of Pf patch on ex vivo human skin. (**A**) Cumulative permeation of Pf from patch and gel vehicles (mean ± SD, *n* = 5). (**B**) Pf flux from patch and gel vehicles.

Effective dosing of Pf in the skin was proportional to the initial drug loading in the patch. Matrix loading of 5 wt% resulted in cumulative permeation of 51.4 ± 9.8 μg/cm^2^ (1 day), 120.2 ± 18.3 μg/cm^2^ (2 days), and 186.0 ± 22.8 μg/cm^2^ (3 days) (Figure 5A). Matrix loading of 15 wt% resulted in cumulative permeation of 129.5 ± 30.2 μg/cm^2^ (1 day), 319.3 ± 82.7 μg/cm^2^ (2 days), and 507.4 ± 109.9 μg/cm^2^ (3 days). The 15 wt% Pf patch performed better than KitosCell^®^, an existing commercial gel formulation used for comparison in both cumulative permeation and flux durability (Figure 5B). Matching the total Pf amount in the donor chamber between the gel and patch vehicles, the 15 wt% patch had a steady state interval from 18–72 h with an average flux of 7.93 μg/cm^2^/h while KitosCell^®^ had a steady state interval from 12–48 h with an average flux of 6.49 μg/cm^2^/h. Furthermore, the patch maintains constant rate of Pf permeation after the initial lag period, while the gel formulation peaked around 24 h and continues to decline in flux over 72 h.

### 3.5. Stability Assessment

**Figure 6:**
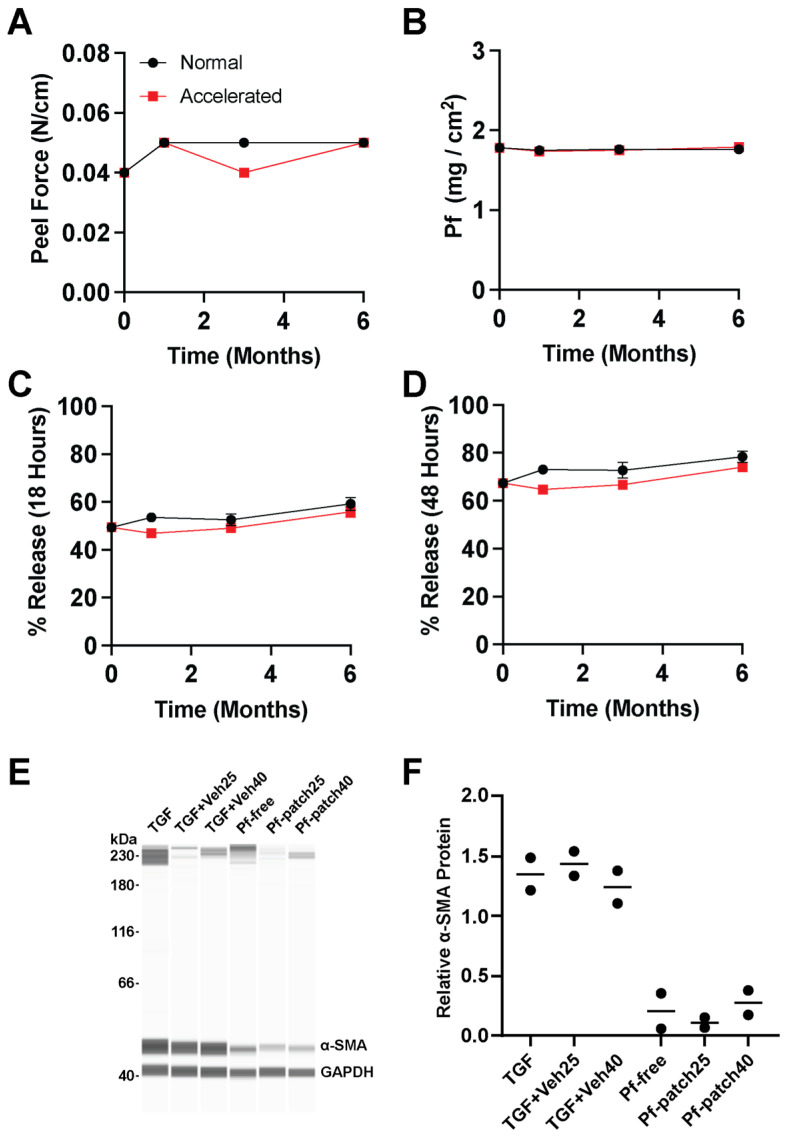
Stability assessment of patches stored under normal (25 °C at 60% RH) and accelerated conditions (40 °C at 75%). (**A**) SSA peel force, (**B**) drug loading of Pf, (**C**) % release of Pf at 18 h (mean ± SD, *n* = 6), (**D**) % release of Pf at 48 h (mean ± SD, *n* = 6), (**E**,**F**) in vitro activity of released Pf in stimulated human dermal myofibroblasts after 6 months of storage at normal (Pf-patch25) or accelerated (Pf-patch40) conditions (mean ± SD, *n* = 2).

Pf patches produced manually in the laboratories using the revised laboratory patch production (Section 2.12) were subject to stability evaluation for the primary performance criteria, including microbial growth, residual solvents, adhesive performance, drug content, release kinetics, and biological activity. Patches stored at 25 °C at 60% RH (normal conditions) for 6 months (Figure 6) and up to 1 months (Supplemental Table S2) and at 40 °C at 75% RH (accelerated conditions) for 6 months were evaluated (Figure 6). For patches stored under normal conditions, no microbial growth was observed on any of the patches evaluated (T0 and T6; Supplemental Table S1). Furthermore, little to no residual solvents were detected in any of the samples, indicating that the drying parameters used for preparation of the drug matrix were effective. Within the study period, no cold flow of either DURO-TAK 87-2852 or MG 7-9850 was observed across all samples (Supplemental Table S1). Peel force of MG 7-9850 was stable throughout the storage period, with a measured force of 0.04 N/cm after initial production and 0.05 N/cm after 6 months of storage at both normal and accelerated conditions (Figure 6A). Pf demonstrated no significant loss or degradation of drug from the patch with 1.78 mg/cm^2^ loading measured initially and after 6 months of storage at accelerated conditions (Figure 6B) with no drug crystallization observed (Supplemental Table S2). While stored Pf patches showed slight increases in the rate of drug release, the drug release profile was generally in line with fresh preparations. Freshly prepared patches showed 49% release at 18 h and 67% release at 48 h, while samples stored at accelerated conditions for 6 months showed 56% release at 18 h and 74% release at 48 h (Figure 6C,D). Pf released from patches and assayed in vitro demonstrated that Pf maintained its biological activity at reducing α-SMA protein expression in TGF-β1-stimulated human dermal fibroblasts after storage for 6 months under accelerated and normal test conditions, respectively (Figure 6E,F). The vehicle did not affect TGF-β1-induced α-SMA protein expression level, and all three Pf samples showed a statistically significant reduction in α-SMA protein expression (One-Way ANOVA, Bonferroni post-test). Furthermore, samples stored for 12 months under normal conditions continued to maintain biological activity (Supplemental Figure S4), drug content, and release kinetics (Supplemental Table S2).

### 3.6. Pilot Scale Production

**Figure 7:**
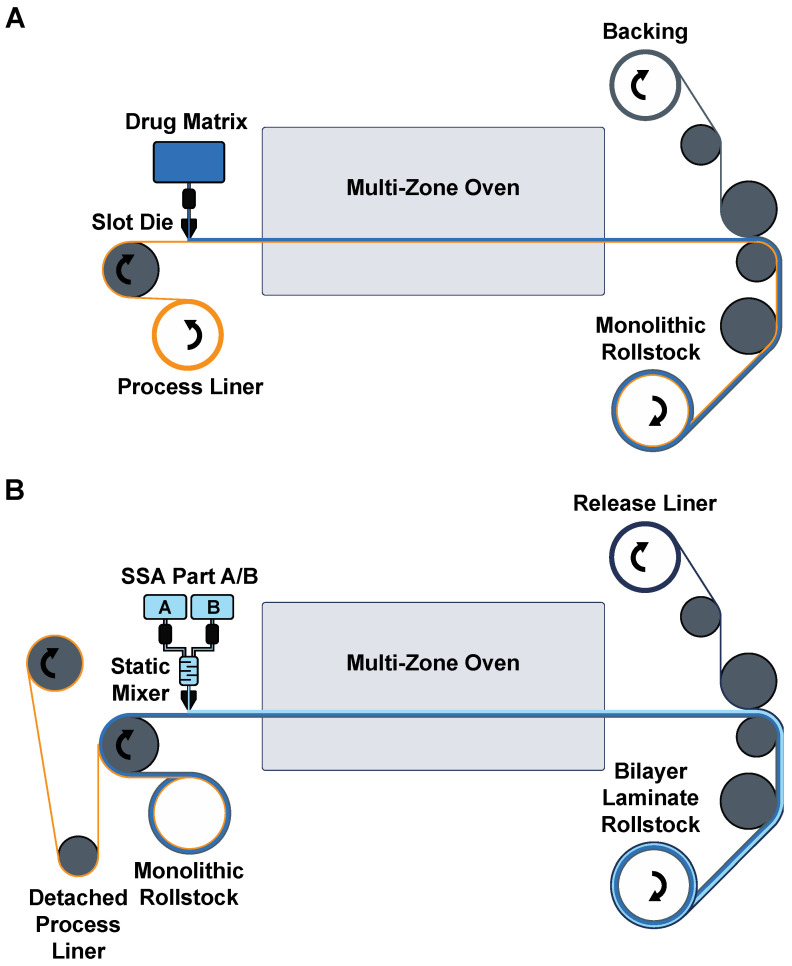
Schematic of pilot scale production of SSA patches. (**A**) Roll-to-roll process for generating standard monolithic patch intermediate. (**B**) Roll-to-roll process for SSA addition and generation of the final patch roll stock.

After reformulation of the patch in laboratory scale with a premade PU backing in preparation of the pilot scale production (Section 2.12), we transitioned to machine-driven automated roll-to-roll process for pilot-scale production of Pf patches (Section 2.15). To mechanically support the thin and flexible PU backing during the coating, drying, and lamination steps, a carrier foil was found to be necessary. Many of the commercially available backing/carrier combinations that we tested were not compatible with the solvents present in DURO-TAK 87-2852 or did not exhibit sufficient mechanical strength or cohesion, resulting in warping, wrinkling, rupture, and lifting of the backing from the carrier layer during a direct coating process (data not shown). Due to these observations, the retention of the paper carrier used for the revised laboratory scale process, as well as the use of siliconized PET carriers, had to be discarded. A BOPP carrier was found to be suitable to generate a wrinkle-free homogeneous bilayer product. Due to the thermosensitivity of this material observed in the preliminary trials at different temperatures, the dryer temperature was limited to 110 °C at maximum. During further process development, emphasis was placed on achieving sufficient crosslinking of the SSA (curing) despite limited drying temperatures. A homogeneous monolayer coating appearance, as well as a low content of residual solvents (<1%), were essential for ensuring good curing. An overview about the final developed pilot scale process is shown in Figure 7.

## 4. Discussion

Patches are planar devices that provide several advantages over spreadable ointments, gels, or creams. They are dry vehicles and do not lose drug to secondary contact, and they are adherent so they do not require secondary dressing or tapes which can improve patient compliance through ease of application, particularly for nursing staff in geriatric or palliative care settings [19]. Patches can be worn over multiple days for uninterrupted delivery or simply removed to terminate dosing. As a vehicle category, patches have had one of the highest translation efficiency and clinical success rates but could pose a high technical barrier for production [18]. As of 2021, only 45 transdermal and dermal patches were available in the US market, but transdermal patches comprise the largest market share, particularly hormone transdermal delivery systems. Greater adoption of patch products for the treatment of skin disease or injury may significantly benefit from practical advantages of patch-based treatment. The adhesive selected for attachment will be a primary determinant of patch performance, particularly in developing a patch for application on compromised skin. Here, we present the laboratory development and pilot-scale processes for soft skin adhesive (SSA) layer addition to the standard monolithic patch design, enabling the direct application of drug-loaded patches over burns for continuous multiday dosing of Pf for treatment of hypertrophic burn scars. Patches can be a lightweight and easily deployable drug vehicle to modulate burn wounds sustained on the battlefield. Additionally, these patches could potentially apply to surgical incision or excision wounds for improved scar outcomes under definitive care.

SSAs are a class of silicone elastomers that are safe on wounds [23,28]. With gentle pressure, SSAs can quickly conform to the texture of skin for secure attachment but will also de-bond under low peel force for a painless and atraumatic removal. Consistent with what has been reported on SSAs [29,30], our own assessment shows that the addition of SSA to the standard monolithic patch we formulated was advantageous to acrylic PSA for reduced skin stripping. While the silicone material was not a suitable drug matrix for Pf loading due to low solubilization, we found that the silicone material (SSA layer) did not negatively impact the drug release kinetics or biological activity of Pf loaded in DURO-TAK adhesive matrix. SSAs have popularly been incorporated into advanced wound care dressings that can be directly attached over the wound bed without the need for secondary tapes or wraps. For the management of burns, SSA products have resulted in positive clinical outcomes in healing and scar prevention of injured skin [31,32,33].

The three critical steps for SSA processing that we highlighted were (1) effective mixing of the two-part adhesive, (2) direct coating on the overlying layer, and (3) high-temperature curing. At the laboratory scale, dual asymmetric centrifugation is an effective process for quick and bubble-free mixing of the two-part curing system. Alternatively, a magnetic stirrer with an additional degassing step can be used. For the semicontinuous pilot scale process, a continuous feed of the two parts into a static mixer has been implemented. With a limited pot-life (30 min at 25 °C), the mixed SSA are then immediately coated over the drug matrix and cured at high temperature to ensure effective bonding and prevent delamination after wear. Moreover, this eliminates the need for cost-intensive fluorosilicone release liners that are otherwise required as a substrate for independent curing of the SSA layer. Due to low peel force and surface free energy of cured SSA, they are compatible with alternate release liners such as low-cost low-density polyethylene (LDPE). The selection of process liners will also be critical for the production process. In the laboratory-scale production scheme, we found that a paper carrier for the backing was the most resistant to warping from the harsh processing conditions of both solvent-based casting solutions and the high temperatures used for solvent evaporation and SSA curing. However, in pilot-scale manufacturing, where higher tensile forces act on the liner due to the web tension between the rollers, a BOPP carrier was best suited to enable the production of a wrinkle-free homogeneous bilayer.

For medical product manufacturing, one of the challenges is transferring the R&D laboratory results (e.g., formulations developed by manual processing in laboratories) to pilot-scale production and finally to the production scale. Both pilot- and large-scale production are largely driven by automated manufacturing processes, which are different from manual processes done in the R&D laboratories. This transition, which requires adaptation and optimization steps, is essential for downstream advanced product development and commercialization for product quality assurance and scale-up production. Here, we showed that direct coating of Pf-drug matrix onto pre-formed PU backing layer, which bypassed the PU coating step as in the manual laboratory fabrication process, enabled pilot-scale manufacturing of drug-in-matrix Pf patches that were comparable to the laboratory patches regarding drug content uniformity and area weight with levels of residual solvent in accordance with ICH guidelines.

Using a patch vehicle, we are interested in the drug delivery of Pf into burn wounds to prevent and mitigate burn wound-associated hypertrophic scarring for improved injury outcome. Pf from patches readily permeates through intact and burned porcine skin, showing comparable cumulative permeation profiles as Pf formulated in semi-solid vehicles [16,34]. In a direct comparison with the commercially available KitosCell^®^ gel, our Pf patch shows improvements in steady state flux when controlling for the total applied dose. As prolonged and excessive inflammation promote scarring [35], the ability of Pf patches at reducing pro-inflammatory markers in porcine burn wounds provides early promise of Pf as a prophylactic treatment to prevent scarring. Further studies are necessary to determine the optimum dosing regimen and treatment schedule for Pf in burns to impart a therapeutic effect on scarring.

Our stability assessment demonstrated that both Pf and the patch vehicle effectively retained their form and functions over 12 months at 25 °C and 60% RH. Adhesive failure is one of the primary modes of interrupting drug dosing. Without effective contact with the skin, the patch is unable to deliver the drug. SSA in our patch formulations maintained its physical properties over time with no indication of cold flow or loss of adhesive strength after prolonged storage. An additional critical failure mode for patch products is drug crystallization which can have detrimental effects on release profile and biological activity, resulting in recall of multiple patch products on the market [36]. By maintaining Pf loading content below our measured saturation solubility of our drug matrix, we did not observe any drug crystallization within our stored patches after 6 months of accelerated storage condition and 12 months of long-term normal storage condition. Furthermore, Pf drug content and biological activity were preserved throughout the stability assessment. Neither the solvent-based patch formulation processes, high temperature layer drying/curing, nor extended storage at 40 °C/75% RH diminished the biological activity of Pf. These results are consistent with reports pointing out the robustness of Pf as a small molecule drug with minimal breakdown under forced degradation conditions [37].

The identification of a dosage formulation in this study supports subsequent studies to determine the optimum dosing regimen and treatment schedule, the sterilization method, dermal safety, and efficacy trials of the Pf patches that could move the drug forward into advanced development as an anti-scar preventative and treatment for improved wound outcomes (e.g., scarring) for warfighters and any burn wound patient.

## 5. Conclusions

We present the laboratory- and pilot-scale production process of a topical patch with an SSA skin contacting layer for direct delivery of Pf into burn wounds. We highlight specific processing requirements for effective assembly of SSA within a drug-in-matrix design. This wound safe patch demonstrates high loading of Pf with an extended-release profile. The patch platforms show constant permeation over time, superior to traditional semi-solid platforms. Additionally, the patch platforms present the advantages of a dry adhesive system that is easy to apply and safe to remove from burn wounds resulting in reduced care burden and increased patient compliance. Furthermore, our SSA patch demonstrates excellent shelf-life regarding drug content, cold flow, release kinetics, biological activity, and adhesive stability.

## Figures and Tables

**Table 1:** Saturation solubility of Pf in commercially available adhesives.

Matrix Material	API (wt%)	Production	Start	1 Week	2 Weeks	3 Weeks	4 Weeks
DURO-TAK 87-2852	20	-	+	++	++	++	++
	18	-	+	++	++	++	++
	16.5	-	-	+	+	+	+
	15	-	-	-	-	-	-
	13.5	-	-	-	-	-	-
	12	-	-	-	-	-	-
GELVA GMS 9073	15	-	-	-	-	+	+
	12.5	-	-	-	-	-	-
	10	-	-	-	-	-	-
DURO-TAK 87-2074	15	-	+	++	++	++	++
	12.5	-	-	-	-	-	-
	10	-	-	-	-	-	-
DURO-TAK 87-2052	15	+	*	*	*	*	*
	12.5	-	+	++	++	++	++
	10	-	-	-	-	-	-
DURO-TAK 87-2516	15	+++	*	*	*	*	*
	12.5	+++	*	*	*	*	*
	10	++	*	*	*	*	*
LIVEO MG 7-9850	1	+++	*	*	*	*	*

(-) No crystallization, (+) Single crystal, (++) Multiple crystals, (+++) Completely crystallized, (*) No storage.

## Data Availability

The data presented are available from the corresponding author upon request.

## Supplementary Materials

The following supporting information can be downloaded at: https://www.mdpi.com/article/10.3390/pharmaceutics15071842/s1, Figure S1: SSA delamination and incomplete curing dependent on effective area weight of SSA layer. (A) Photograph of patch variations applied to the back of the glove. (B) Photograph of back of the glove after patch removal demonstrating SSA layer failure. (C) Effective area weight of the SSA layer across the drug matrix thicknesses and SSA layer thicknesses patch variations. Figure S2: Area weight and Pf loading characterization of patches used in in vivo and ex vivo porcine studies. (A) Area weight of patch vehicle controls across four batches (*n* = 150). (B) Area weight of pirfenidone loaded patches across four batches (*n* = 181). (C) Drug loading quantification of pirfenidone loaded patches across four batches (*n* = 150). Box and whisker plots with whiskers showing 10–90 percentile. Figure S3: In vitro permeation test (IVPT) through intact human skin of patches with (A) varying thickness of commercial backing and (B) varying thickness of MG 7-9850 SSA layer. Figure S4: In vitro activity of released Pf in stimulated human dermal myofibroblasts after 12 months of storage at normal conditions (25 °C at 60% RH). (mean ± SD, *n* = 2). Table S1: List of primer sets for in vivo RNA gene expression. Table S2: 12-month stability assessment of patches (25 °C at 60% RH) or 6-month assessment of patches (40 °C at 75% RH) for cold flow, crystallization, area weights, % drug content, residual solvents, and microbial quality. n.a. = Not available.
