# Supplementary material for: A Soft Skin Adhesive (SSA) Patch for Extended Release of Pirfenidone in Burn Wounds
Source: Pharmaceutics. 2023 Jun 28;15(7):1842. doi: 10.3390/pharmaceutics15071842 (PMC10386754; doi:10.3390/pharmaceutics15071842)
Supplement: Supplementary file 1 [file pharmaceutics-15-01842-s001.zip › pharmaceutics-2459137-supplementary.pdf]

Supplemental Materials

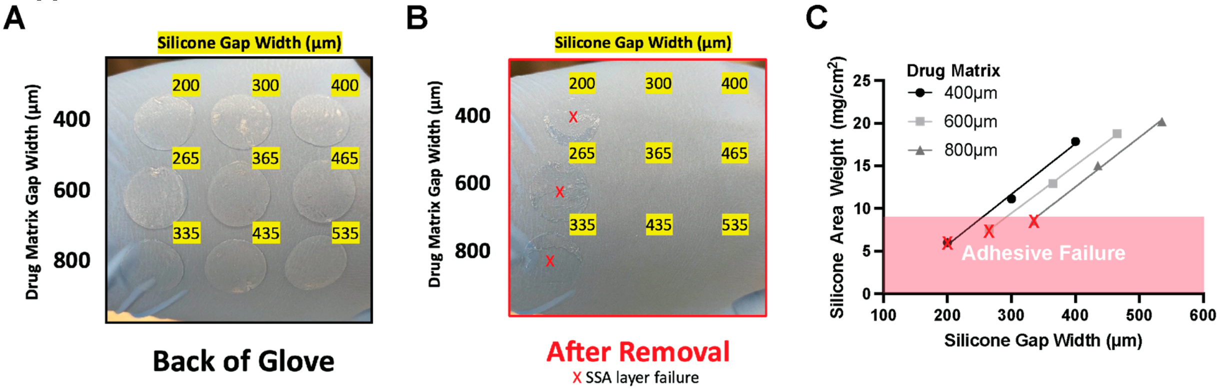

Supplemental Figure S1. SSA delamination and incomplete curing dependent on effective area weight of SSA layer. (A) Photograph of patch variations applied to the back of the glove. (B) Photograph of back of the glove after patch removal demonstrating SSA layer failure. (C) Effective area weight of the SSA layer across the drug matrix thicknesses and SSA layer thicknesses patch variations.

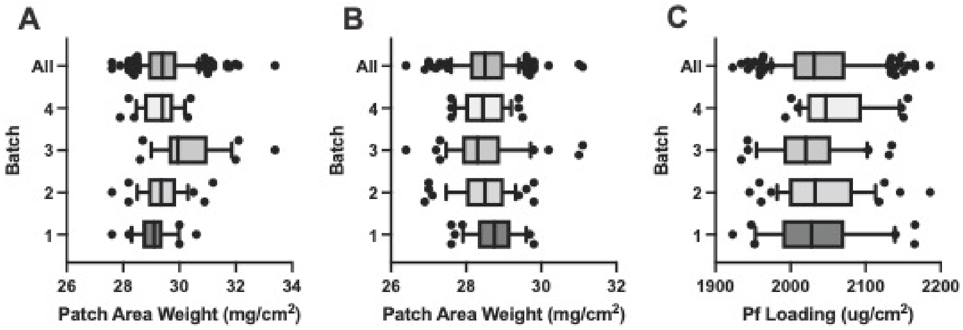

Supplemental Figure S2. Area weight and Pf loading characterization of patches used in in vivo and ex vivo porcine studies. (A) Area weight of patch vehicle controls across four batches (n=150). (B) Area weight of pirfenidone loaded patches across four batches (n=181). (C) Drug loading quantification of pirfenidone loaded patches across four batches (n=150). Box and whisker plots with whiskers showing 10-90 percentile.

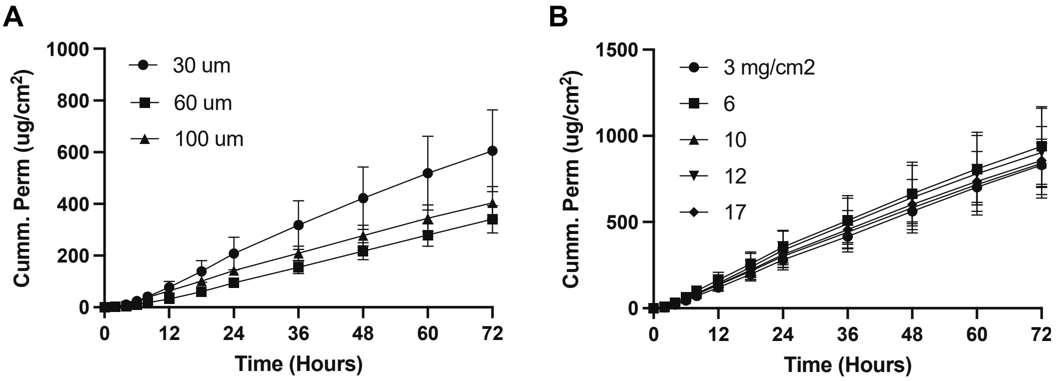

Supplemental Figure S3. *In vitro* permeation test (IVPT) through intact human skin of patches with (A) varying thickness of commercial backing and (B) varying thickness of MG 7-9850 SSA layer.

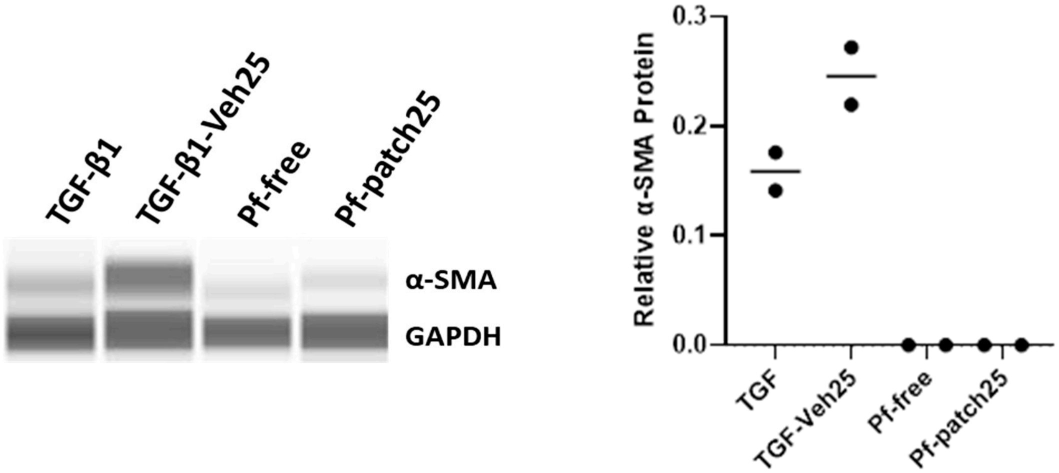

Supplemental Figure S4. In vitro activity of released Pf in stimulated human dermal myofibroblasts after 12 months of storage at normal conditions (25ºC at 60% RH). (mean ± SD, n=2).

Supplemental Table S1. List of primer sets for *in vivo* RNA gene expression.

Primer	Sequence 5' to 3'
MIP-1α F	TCCTCGCAAATTCGTAGCCG
MIP-1α R	TCAGCTCCAGGTCAGAGATGT
IL-8 F	AGTGCAGAACTTCGATGCCA
IL-8 R	AGCCACGGAGAATGGGTTTT
IL-1β F	GGTACATGGTTGCTGCCTGA
IL-1β R	TGGCTTAGAGGATGGGGTCT
IL-6 F	GGTGATGCCACCTCAGACAA
IL-6 R	TCTGCACAGCCTCGACATTT
IL-17A F	GCCACTCGGGCTGTATCAAT
IL-17A R	GGATCTCTTGCTGGATGGGG
IL-24 F	CGCAGGCGTTTTCTGCTATT
IL-24 R	TCAGCGCTGGTAGAATGTCTC
MMP-9 F	CCAGCACAGACGAGTTCCTT
MMP-9 R	GTCACCCCCAAAACCATTGC
MMP-13 F	AGCCGTTCACTTTGAGGCTA
MMP-13 R	CTCTCCCTCCCTAGCAACTCC
TGFB1 F	ATTTAAGGACATCGTGCCCCA
TGFB1 R	CCGCACCTGAGACATATGGA
MTF F	AGTCGTTGATAGCTCCTGTCG
MTF R	AGATGGGAGGGGACAACCAA

Supplemental Table S2. 12-month stability assessment of patches (25 ºC at 60% RH) or 6-month assessment of patches (40 ºC at 75% RH) for cold flow, crystallization, area weights, % drug content, residual solvents, and microbial quality. n.a. = Not available.

		**Initial**		**1 Month**		**3 Month**		**6 Month**		**12 Month**
Parameter	Units	25ºC / 60% RH	40ºC / 75% RH	25ºC / 60% RH	40ºC / 75% RH	25ºC / 60% RH	40ºC / 75% RH	25ºC / 60% RH	40ºC / 75% RH	25ºC / 60% RH
Cold Flow		0	0	0	0	0	0	0	0	0
Crystals		not visible	not visible	not visible	not visible	not visible	not visible	not visible	not visible	not visible
Weight										
Mean	mg	200.6	200.6	195.8	197.5	197.9	198.7	197.1	194.4	196.4
Min	mg	199.6	199.6	192.6	195.6	195.4	197	196.4	192.5	194.6
Max	mg	201.5	201.5	200.8	200.4	200.3	200	197.7	196.4	198.3
Drug Content	mg / 5cm^2	8.90	8.90	8.74	8.68	8.81	8.74	8.80	8.94	8.71
% Drug Content of Label	%	98.9	98.9	97.1	96.5	97.9	97.1	97.8	99.3	96.8
18 Hour Drug Release	%	49 ± 2	49 ± 2	54 ± 1	47 ± 1	53 ± 2	49 ± 1	59 ± 3	56 ± 2	56 ± 2
48 Hour Drug Release	%	67 ± 2	67 ± 2	73 ± 1	65 ± 1	63 ± 3	67 ± 2	78 ± 2	74 ± 2	74 ± 2
Residual Solvents										
Ethyl Acetate	ppm	2	2	n.a.	n.a.	n.a.	n.a.	n.a.	n.a.	n.a.
2-Propanol	ppm	18	18	n.a.	n.a.	n.a.	n.a.	n.a.	n.a.	n.a.
n-Hexane	ppm	n.d.	n.d.	n.a.	n.a.	n.a.	n.a.	n.a.	n.a.	n.a.
Toluene	ppm	n.d.	n.d.	n.a.	n.a.	n.a.	n.a.	n.a.	n.a.	n.a.
Acetylacetone	ppm	47	47	n.a.	n.a.	n.a.	n.a.	n.a.	n.a.	n.a.
Microbiological Quality		free of microorganisms	free of microorganisms	n.a.	n.a.	n.a.	n.a.	free of microorganisms	free of microorganisms	free of microorganisms
